# Biomarker-Driven Optimization of Saponin Therapy in MASLD: From Mouse Models to Human Liver Organoids

**DOI:** 10.3390/antiox14080943

**Published:** 2025-07-31

**Authors:** Hye Young Kim, Ju Hee Oh, Hyun Sung Kim, Dae Won Jun

**Affiliations:** 1 Hanyang Institute of Bioscience and Biotechnology, Hanyang University, Seoul 04763, Republic of Korea; kkimhy0927@hanyang.ac.kr; 2 Department of Internal Medicine, College of Medicine, Hanyang University, Seoul 04763, Republic of Korea; 3 Department of Obstetrics and Gynecology, Institute of Women’s Medical Life Science, Yonsei Cancer Center, Severance Hospital, Yonsei University College of Medicine, Seoul 03722, Republic of Korea; onk1210@yuhs.ac; 4 Department of Pathology, College of Medicine, Hanyang University, Seoul 04763, Republic of Korea

**Keywords:** metabolic dysfunction-associated steatotic liver disease, red ginseng, saponin, biomarker, HAMP1, patient-derived liver organoid

## Abstract

(1) Background: Metabolic dysfunction-associated steatotic liver disease (MASLD) is characterized by liver damage similar to alcoholic fatty liver disease, including triglyceride infiltration of hepatocytes, regardless of alcohol consumption. It leads to progressive liver damage, such as loss of liver function, cirrhosis, and liver cancer, and the response rate of drugs under clinical research is less than 50%. There is an urgent need for biomarkers to evaluate the efficacy of these drugs. (2) Methods: MASLD was induced in mice using a High-Fat diet (HF), Western diet (WD), and Methionine/Choline-Deficient diet (MCD) for 20 weeks (4 weeks for MCD). Liver tissue biopsies were performed, and the treatment effects of saponin and non-saponin feeds were evaluated. Fat accumulation and hepatic inflammation were measured, and mRNA sequencing analysis was conducted. The therapeutic effects were validated using patient-derived liver organoids. (3) Results: The NAFLD Activity Score (NAS) significantly increased in all MASLD models compared with controls. Saponin treatment decreased NAS in the HF and WD groups but not in the MCD group. RNA sequencing and PCA analysis showed that the HF saponin response samples were similar to normal controls. DAVID analysis revealed significant changes in lipid, triglyceride, and fatty acid metabolic processes. qRT-PCR confirmed decreased fibrosis markers in the HF saponin response group, and GSEA analysis showed reduced *HAMP1* gene expression. (4) Conclusions: Among the diets, red ginseng was most effective in the HF diet, with significant effects in the saponin-treated group. The therapeutic efficacy was better when *HAMP1* expression was increased. Therefore, we propose *HAMP1* as a potential exploratory biomarker to assess the saponin response in a preclinical setting. In addition, the reduction of inflammation and hepatic iron accumulation suggests that saponins may exert antioxidant effects through modulation of oxidative stress.

## 1. Introduction

MASLD is a disorder defined by the buildup of triglycerides in the liver and is acknowledged as one of the primary causes of chronic liver conditions globally [1]. In the Republic of Korea, the prevalence of MASLD is reported to be approximately 30%, reflecting a significant public health concern [2]. Despite the increasing prevalence of MASLD, two significant challenges remain. Firstly, there is only one FDA-approved medication available for treating MASLD, highlighting the pressing demand for the creation of additional treatment options [3]. This limitation highlights the necessity for continued research to discover and validate effective treatments. Second, MASLD presents with considerable variability in metabolic characteristics among patients, leading to differences in disease onset, progression, and response to treatment. This variability often results in distinct therapeutic outcomes, emphasizing the need for personalized treatment approaches [4]. Prior studies have demonstrated that red ginseng exhibits hepatoprotective properties, potentially offering therapeutic benefits in the management of hepatic disorders [5,6]. However, it remains unclear whether these effects are due to its saponin components or other non-saponin constituents. To address this gap, our study aimed to investigate the hepatoprotective properties of red ginseng by identifying whether its therapeutic effects are attributable specifically to its saponin components or other non-saponin components. We also assessed which MASLD subtypes would respond most effectively to red ginseng treatment using mouse models induced by three different diets. Moreover, we evaluated potential biomarkers that could predict the efficacy of red ginseng in specific patient subgroups. Through these objectives, this study sought to evaluate the potential of red ginseng as a novel therapeutic agent for MASLD and to advance the understanding of its efficacy in diverse patient populations. Identifying reliable biomarkers for predicting treatment response could pave the way for more personalized and effective management strategies for MASLD. Oxidative stress is a key factor in the pathogenesis and progression of MASLD, contributing to hepatocyte damage through the accumulation of reactive oxygen species (ROS) and lipid peroxidation [7,8]. Red ginseng and its saponin components have been reported to exert antioxidant activities, such as scavenging reactive oxygen species and modulating oxidative stress-related signaling pathways [9,10]. Therefore, we further hypothesized that the hepatoprotective effects of red ginseng may be partly mediated through the attenuation of oxidative stress. Furthermore, considering the role of hepatic iron overload in promoting ROS generation via the Fenton reaction, we evaluated changes in iron metabolism markers, including *HAMP1*, as potential mediators of red ginseng’s antioxidant effect. This oxidative stress-related perspective reinforces the potential relevance of red ginseng as a therapeutic agent for MASLD in the context of redox biology.

## 2. Materials and Methods

### 2.1. Animal Study Design

Five-week-old male C57BL/6J mice (20–22 g) were purchased from Dae Han BioLink Co. (Chungbuk, Republic of Korea). Following a one-week quarantine and acclimation period, the mice were housed under controlled conditions (22 ± 2 °C, 50 ± 15% humidity, 12 h light/dark cycle). A total of 100 mice were used, from which 10 animals were assigned to the normal chow group, and the remaining 90 mice were equally allocated to one of three experimental diet groups: High-fat diet (HFD; Research Diets, Inc., New Brunswick, NJ, USA; D12492, 60 kcal% fat), Western diet (WD; Research Diets, Inc., New Brunswick, NJ, USA; D12079B), or methionine- and choline-deficient diet (MCD; Research Diets, Inc., New Brunswick, NJ, USA; A02082002BR). The diets were administered for 20 weeks (MCD for 4 weeks) to induce fatty liver.

Liver biopsies were performed after the feeding period to confirm MASLD [11]. Each group was then subdivided into three treatment arms (*n* = 10 per subgroup): vehicle control, saponin (200 mg/kg), or non-saponin (200 mg/kg). The saponin and non-saponin components (provided by the Korean Society of Ginseng) were incorporated into the diets at 200 mg/kg (~10 mg/50 g body weight) and administered for an additional 8 weeks.

All animal procedures were conducted in accordance with the Hanyang University Institutional Animal Care and Use Committee guidelines (Approval No. HY-IACUC-20-0043, approved on 28 October 2019).

### 2.2. Liver Biopsy and Randomization

At week 20, partial liver biopsies were performed in mice from the HFD and WD groups to confirm disease induction. Mice were anesthetized using intraperitoneal (i.p.) injections of Zoletil (Virbac, Carros, France) and Rompun (Bayer, Leverkusen, Germany). The abdomen was shaved and sterilized with 10% iodine, and a midline incision (<1 cm) was made. A portion of the left lateral lobe of the liver was then excised. Hemostasis was achieved by applying a heated spatula, and the incision was sutured with 5–0 absorbable sutures. Postoperative care included heat lamp warming and tetracycline treatment for 3 days.

### 2.3. Preparation of Ginsenoside (Saponin) and Non-Saponin Fractions

Red ginseng extract was fractionated using HP20 resin (Mitsubishi Chemical Industries, Ltd., Tokyo, Japan) via adsorption chromatography. The extract was diluted to 10% in water, filtered, and loaded onto the HP20 column. Sequential elution with water, 30% ethanol, and 95% ethanol yielded three fractions. The first two (water and 30% ethanol) were combined and processed into the non-saponin fraction (NS-RG), while the 95% ethanol fraction was used to prepare the saponin fraction (S-RG) via concentration and spray drying. Saponin and non-saponin fractions were dissolved in autoclaved triple-distilled water (TDW) and administered at final concentrations of 0.1 mg/mL and 0.5 mg/mL. For qRT-PCR and Western blot analyses, samples were harvested after 24 h of treatment, whereas for ROS measurements using the DCFDA/H2DCFDA Cellular ROS Assay Kit, fluorescence intensity was assessed 6 h post-treatment.

### 2.4. RNA Sequencing and Data Analysis

Total RNA was extracted and libraries were prepared using the NEBNext Ultra II Directional RNA-Seq Kit (New England Biolabs, Hitchin, UK). Poly(A) RNA was selected using the LEXOGEN kit (Lexogen, Vienna, Austria), followed by cDNA synthesis and fragmentation. Libraries were indexed (Illumina 1–12), enriched by PCR, and quality-checked using the Agilent 2100 Bioanalyzer (Agilent Technologies, Santa Clara, CA, USA). Sequencing was conducted using the Illumina NovaSeq 6000 (Illumina, San Diego, CA, USA) (paired-end 100 bp reads).

Raw reads were quality-checked with FastQC v0.12.1 [12], and low-quality reads (Q < 20) were trimmed using FASTX_Trimmer v0.0.14 and BBMap v39.15 [13]. Reads were aligned to the reference genome with TopHat v2.1.1 [14]. Gene expression was quantified using FPKM values via Cufflinks [15], normalized by quantile normalization (EdgeR v3.42.4, R). Data visualization was performed with ExDEGA v5.2.1 (Ebiogen Inc., Seoul, Republic of Korea).

### 2.5. Histopathological Analysis

Liver tissues were fixed in 10% formalin, embedded in paraffin, sectioned (4–5 μm), and stained with hematoxylin and eosin (H&E). Slides were evaluated under a light microscope, and NAFLD-related features (steatosis, inflammation, ballooning) were scored using the NAFLD Activity Score (NAS).

### 2.6. Blood Biochemical Analysis

Blood samples were centrifuged at 3000 rpm for 15 min at 4 °C to isolate serum. Serum ALT, AST, total cholesterol, and triglyceride levels were analyzed using a Hitachi 7180 automated analyzer (Hitachi Ltd., Tokyo, Japan).

### 2.7. Glucose Tolerance Test (GTT)

Mice were fasted for ≥6 h and received a 50% glucose solution intraperitoneally (dose = 4 × body weight in g). Blood glucose levels were measured at 15-, 30-, 60-, and 120-min post-injection [16].

### 2.8. Quantitative Reverse Transcription PCR (qRT-PCR)

Total liver RNA was extracted using the Trizol method. RNA concentration was measured with a NanoDrop ND-2000 (Thermo Fisher Scientific, Waltham, MA, USA). We reverse transcribed 2 µg of RNA using the Prime-Script RT Reagent Kit (TaKaRa Bio Inc., Shiga, Japan). qPCR was performed on a LightCycler 480 (Roche, Mannheim, Germany) using SYBR Green Master Mix (Thermo Fisher Scientific, Waltham, MA, USA) in 10 µL reactions. Fold changes were calculated relative to control after normalization.

### 2.9. Western Blot Analysis

Liver proteins were extracted using RIPA buffer assay (Thermo Fisher Scientific, Waltham, MA, USA, #89900) supplemented with protease inhibitor(GenDEPOT, Barker, Battle Creek, MI, USA, P3100-001) and phosphatase inhibitors(GenDEPOT, Barker, Battle Creek, MI, USA, P3200-001). After 30 min incubation on ice, lysates were centrifuged (12,000 rpm, 30 min, 4 °C). Proteins were quantified using a BCA assay (Thermo Fisher Scientific, Waltham, MA, USA) and resolved on 4–20% G-cast precast gels (Bio-Rad Laboratories, Hercules, CA, USA, #4561096). Band intensity was quantified using ImageJ v 2.9.0 and normalized to GAPDH or β-actin.

### 2.10. Prussian Blue Staining

Iron deposits in liver tissue were visualized using the Abcam Ferric Iron Detection Kit (Abcam, Cambridge, UK, ab150674), following the manufacturer’s instructions.

### 2.11. ROS Measurement

Intracellular ROS levels were assessed using the DCFDA/H2DCFDA Cellular ROS Assay Kit (Abcam, Cambridge, UK, ab113851) according to the manufacturer’s protocol. Organoids were incubated with 20 µM DCFDA for 45 min at 37 °C in the dark, followed by PBS washing and fluorescence measurement at Ex/Em = 485/535 nm.

### 2.12. Human Liver Organoid Culture

Liver tissue (~1 cm^3^) was obtained from patients undergoing cholecystectomy at Kangbuk Samsung Hospital (IRB No. 2022-10-050, approved on 21 December 2022). After informed consent, tissues were digested using collagenase-trypsin, centrifuged, and resuspended in BME2 (R&D Systems, Minneapolis, MN, USA). Organoids were seeded in 24-well plates (3000–10,000 cells/well).

The culture medium was composed of AdDMEM/F12 (Thermo Fisher Scientific, Waltham, MA, USA) supplemented with 1% Pen/Strep (Thermo Fisher Scientific, Waltham, MA, USA), 10 mM HEPES (Thermo Fisher Scientific, Waltham, MA, USA), 1% GlutaMax (Thermo Fisher Scientific, Waltham, MA, USA), 1% N2 (Thermo Fisher Scientific, Waltham, MA, USA), 1% B27 (Thermo Fisher Scientific, Waltham, USA), 1.25 mM N-Acetylcysteine (Sigma-Aldrich, St. Louis, MO, USA), 10 nM gastrin (Sigma-Aldrich, St. Louis, MO, USA), and the following growth factors: 50 ng/mL EGF (PeproTech, Rocky Hill, CT, USA), 250 ng/mL R-spondin 1 (PeproTech, Rocky Hill, CT, USA), 50 ng/mL FGF10 (PeproTech, Rocky Hill, USA), 25 ng/mL HGF (PeproTech, Rocky Hill, CT, USA), 10 mM nicotinamide (Sigma-Aldrich, St. Louis, MO, USA), 5 µM A83-01 (Tocris Bioscience, Bristol, UK), 25 ng/mL Noggin (PeproTech, Rocky Hill, CT, USA), 10 µM forskolin (Sigma-Aldrich, St. Louis, MO, USA), 10 µM Y-27632 (Tocris Bioscience, Bristol, UK), and 50 µg/mL Primocin (InvivoGen, San Diego, CA, USA) [17].

### 2.13. LipidTOX Staining

Liver organoids were embedded in BME (40 µL) in LabTek chambers (Thermo Fisher Scientific, Waltham, MA, USA, 154534) and stained with LipidTOX™ Deep Red (Thermo Fisher Scientific, Waltham, MA, USA) (1:200 dilution in PBS) for 30 min at room temperature. Nuclei were counterstained with DAPI-containing mounting solution (Vector, Newark, NJ, USA, H-1500-10). Fluorescence microscopy was used for imaging.

### 2.14. Statistical Analysis

All data are expressed as mean ± SEM. Statistical analysis was performed using GraphPad Prism 8. One-way ANOVA and independent *t*-tests were used to compare groups. Statistical significance is indicated as follows: * *p* < 0.05, ** *p* < 0.01, *** *p* < 0.001, **** *p* < 0.0001. A *p*-value < 0.05 was considered statistically significant. All experiments were performed in triplicate unless otherwise noted.

### 2.15. Chemicals and Reagents

The saponin and non-saponin fractions used in this study were provided by the Korea Ginseng Corporation (KGC). Detailed information regarding the preparation and component analysis is described in Section 2.3.

Other key reagents included the DCFDA/H2DCFDA—Cellular ROS Assay Kit (Abcam, Cambridge, UK, ab113851) for measuring ROS, the LipidTOX™ Deep Red Neutral Lipid Stain for cellular imaging (Thermo Fisher Scientific, Waltham, MA, USA, H34477) and assessing intracellular lipid accumula-tion, and the Ferric Iron Detection Kit (Prussian Blue Staining; Abcam, Cambridge, UK, ab150674) for visualizing hepatic iron deposits.

Reagents used for qRT-PCR and Western blot analyses included TRIzol™ reagent (Invitrogen, San Diego, CA, USA), RIPA buffer (Thermo Fisher Scientific, Waltham, MA, USA), the BCA Protein Assay Kit (Thermo Fisher Scientific, Waltham, MA, USA), and SYBR Green Master Mix (Roche, Mannheim, Germany).

## 3. Results

### 3.1. Comparison of Metabolic Dysfunction Associated Steatotic Liver Disease Mouse Models Induced by Three Different Diets

**Figure 1:**
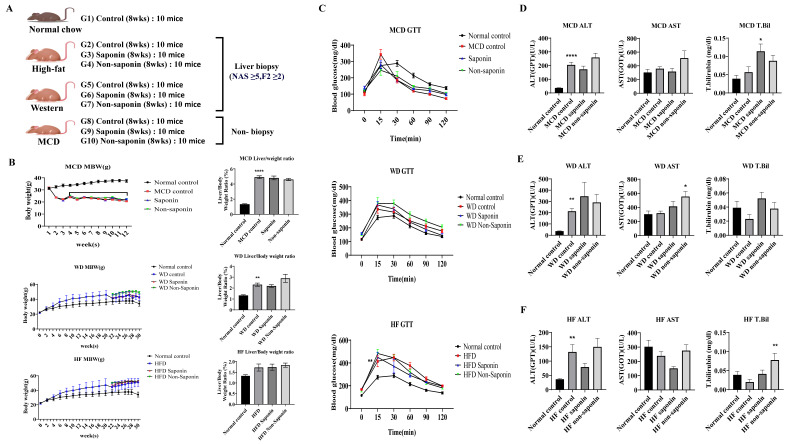
Overview of MASLD diet-induced mouse models and liver injury markers. (**A**) Experimental schedule outlining the feeding and treatment protocol for the MASLD 3 diet-induced groups. (**B**) Body weight and liver-to-body weight ratio in the MASLD diet-induced groups. (**C**) Glucose tolerance test (GTT) results (mg/dL) in the MASLD diet-induced groups. (**D**–**F**) Serum levels of alanine aminotransferase (ALT, U/L), aspartate aminotransferase (AST, U/L), and total bilirubin (mg/dL) in (**D**) the Methionine–Choline-Deficient (MCD) diet group, (**E**) the Western diet group, and (**F**) the High-Fat diet group. Data are presented as mean ± SEM. Statistical analysis was performed using one-way ANOVA followed by Tukey’s post-hoc test. Non-significant comparisons are not labeled. Statistical significance is indicated by asterisks: * *p* < 0.05, ** *p* < 0.01, **** *p* < 0.0001.

To establish metabolic dysfunction-associated steatotic liver disease (MASLD) models, mice were fed with WD or HFD for 20 weeks to induce fatty liver. The MCD diet was administered for 4 weeks. After diet-induced induction of fatty liver, animals with an NAS ≥ 3 (in HFD and WD models) were selected for further analysis. These mice were then administered saponin or non-saponin fractions for 8 weeks, as outlined in the experimental design (Figure 1A) [3].

All three inflammation groups showed a significant increase in liver/weight ratio compared with the normal control, but no statistically significant differences were observed between the saponin and non-saponin treatment groups within each inflammation model (Figure 1B). Glucose tolerance tests (GTT) conducted on the three inflammation models revealed no significant differences compared with the normal control (Figure 1C).

Biochemical analysis was performed on blood obtained from endpoint biopsies. In the MCD group, ALT levels were significantly elevated in the MCD control compared with the normal control, and a decreasing trend was observed in the MCD saponin-treated group. However, the total bilirubin level was significantly higher in the MCD saponin group compared with the MCD control (Figure 1D). In the Western diet group, ALT levels were significantly increased in the Western control compared with the normal control, and both saponin and non-saponin treatment groups also showed increasing trends (Figure 1E). Similarly, in the High-Fat diet group, ALT levels were significantly higher in the HF control compared with the normal control, with a decreasing trend observed in the saponin-treated group (Figure 1F).

### 3.2. NAFLD Activity Score and Histological Analysis of Liver Tissue in the Three Inflammation Models

**Figure 2:**
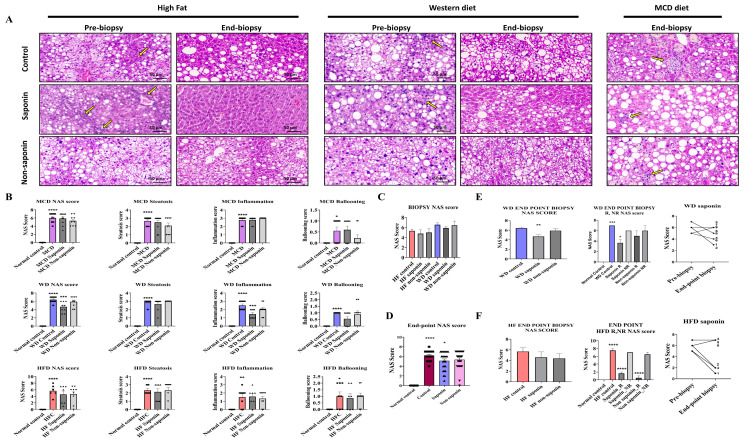
Histological and scoring assessment of MASLD progression and treatment response. (**A**) Representative hematoxylin and eosin (H&E) images from the MASLD 3 diet-induced groups. Yellow arrows indicate inflammatory cell infiltration in hepatic lobules. Scale bar = 50 µm. (**B**) Total NAFLD Activity Score (NAS) and its components—steatosis, inflammation, and ballooning—at endpoint liver biopsies across the three MASLD diet groups. (**C**) Pre-biopsy NAS scores in mice fed High-Fat (HF) or Western diet (WD), treated with control, saponin, or non-saponin. Data are presented as mean ± SEM; n = 10 mice per group. (**D**) Endpoint NAS scores in normal, control, saponin-, and non-saponin-treated groups. (**E**) Western diet group: endpoint NAS scores, stratification by responder vs. non-responder, intra-group change in NAS, and pre- vs. post-biopsy NAS comparison. (**F**) High-fat diet group: endpoint NAS scores, stratification by responder vs. non-responder, intra-group change in NAS, and pre- vs. post-biopsy NAS comparison. Statistical significance is indicated by asterisks: * *p* < 0.05, ** *p* < 0.01, *** *p* < 0.001, **** *p* < 0.0001.

Liver tissues from the three MASLD models were analyzed using H&E staining to assess the NAS, which integrates steatosis score, hepatocyte ballooning, and lobular inflammation score (Figure 2A). It was observed that NAS was significantly elevated in the High-Fat diet control, WD control, and MCD diet control groups compared with the normal control (Figure 2B). When analyzing the combined data from the three inflammation models, a significant reduction in NAS was observed in the saponin-treated groups compared with the MASLD control mice (Figure 2C). Sirius Red staining indicated a tendency towards reduced fibrosis area in the saponin and non-saponin response groups compared with the High-Fat diet control group (Figure 2D).

In the Western diet group, the NAS was significantly reduced in the saponin-treated group compared with the control (*p* = 0.0037) (Figure 2E). Although a decreasing trend in NAS was observed in both saponin and non-saponin treated groups in the High-Fat diet model, the reduction was not statistically significant. However, a comparative analysis of NAS changes revealed a greater reduction in the NAS for the saponin-treated group in the High-Fat diet (3.666 points) compared with that in the Western diet (2 points).

By comparing the NAS from the pre-biopsy and endpoint biopsy period, samples were classified as response if there was improvement and non-response if there was deterioration. The analysis showed that the saponin response in the High-Fat diet group exhibited a higher significance (*p* < 0.0001) compared with the Western diet saponin response (*p* = 0.0103) (Figure 2F). Consequently, although the percentage reduction was higher in the Western diet group, the reduction rate of the NAS was more substantial in the High-Fat diet group.

### 3.3. NAS and Molecular Analysis in Liver Tissue

The NAS from pre-biopsies and endpoint biopsies were compared, with samples showing improvement classified as response and those deteriorating classified as non-response. The results indicated that the saponin response in the High-Fat diet group exhibited greater statistical significance (*p* < 0.0001) compared with the saponin response in the Western diet group (*p* = 0.0103) (Figure 2E,F). Thus, while the percentage reduction was higher in the Western diet group, the rate of NAS reduction was more pronounced in the High-Fat diet group. The three diets used to induce MASLD exhibited distinct differences [18]. The HFD promoted lipid accumulation in hepatocytes and triggered insulin resistance and obesity, mirroring the early metabolic characteristics of MASLD [19,20]. The Western diet not only results in lipid accumulation but is also accompanied by severe inflammatory responses and fibrosis. The MCD diet, by directly disrupting hepatic lipid metabolism due to the absence of methionine and choline, induces MASLD [21]. This diet leads to severe steatosis, inflammation, fibrosis, and hepatocyte injury without significant weight gain.

This study aimed to identify the dietary models in which saponins and non-saponins show optimal efficacy. In both the MCD and Western diet models, fibrosis and inflammation markers increased, while the anti-fibrosis marker MMP9 decreased with saponin treatment. Although some markers showed trends of improvement, such as reduced SREBP1c in the Western diet model, no statistically significant therapeutic effects were observed between saponin and non-saponin treatments. Overall, neither treatment demonstrated significant efficacy in improving fibrosis or inflammation in the MCD or Western diet models (Figure S1).

### 3.4. mRNA Expression and Protein Analysis in Liver Tissue

**Figure 3:**
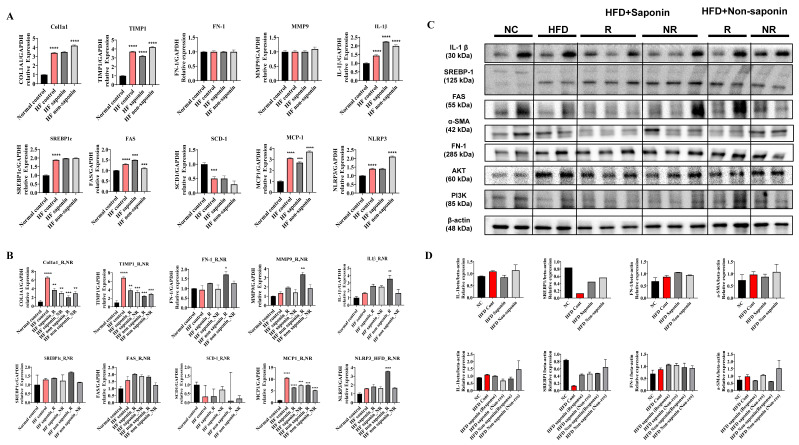
Molecular profiling of fibrosis, inflammation, and lipogenesis markers in the High-Fat diet (HFD) group. (**A**) qRT-PCR analysis of pooled liver tissues from endpoint HFD group mice. Gene expression levels of fibrosis markers (*Col1a1*, *Timp1*, *Fn1*), inflammation markers (*Mmp9*, *Il-1β*, *Mcp-1*, *Nlrp3*), and lipogenesis markers (*Srebp1c*, *Fas*, *Scd-1*) were evaluated. (**B**) Endpoint liver tissues from the HFD group were categorized into saponin response, non-response, non-saponin response, and non-response groups. qRT-PCR was performed to analyze fibrosis, inflammation, and lipogenesis markers across these subgroups. (**C**) Western blot analysis was conducted on endpoint liver tissues from the same categorized groups in the HFD cohort, assessing representative markers of fibrosis, inflammation, and lipogenesis. (**D**) Quantitative densitometry data for the Western blot results are shown as bar graphs. Statistical significance is indicated by asterisks: * *p* < 0.05, ** *p* < 0.01, *** *p* < 0.001, **** *p* < 0.0001.

mRNA expression levels were assessed in liver tissues from the normal control and High-Fat diet groups at endpoint biopsy. The fibrosis marker *TIMP-1* was significantly reduced in the saponin-treated group compared with the High-Fat control. *Col1A1* levels were significantly elevated in the High-Fat control compared with the normal control, but no significant changes were observed in the saponin-treated group. Additionally, no significant differences were found in Fibronectin1 (*FN1*) and *MMP9* levels. Inflammation markers *IL-1β* and *MCP-1* were significantly increased in the High-Fat control group compared with the normal control, with *MCP-1* showing a significant decrease in the saponin-treated group (*p* = 0.0007). However, the lipogenesis marker *SREBP1c* and the VLDL secretion marker *SCD-1* did not show significant differences (Figure 3A; Table 1).

When the High-Fat diet group was categorized into response (N ≤ 3) and non-response (N > 4) based on histology scores, mRNA expression levels were compared. *TIMP-1* and *Col1A1* levels were significantly reduced in both saponin response and non-saponin response groups. Additionally, *IL-1β* was significantly increased in the non-saponin response group, while *MCP-1* was significantly decreased in the saponin response group. However, *SREBP1c* did not show significant differences between saponin and non-saponin treatment groups, and *SCD-1* also showed no significant differences (Figure 3B). Based on these findings, we propose that saponin treatment may be effective in reducing fibrosis.

Subsequent Western blot analysis was performed to evaluate protein levels (Figure 3C; Table 2). IL-1β was increased in the High-Fat control compared with the normal control and decreased in the saponin-treated group compared with the High-Fat control. Specifically, the fibrosis marker α-SMA was elevated in the High-Fat control compared with the normal control and decreased in both saponin response and non-saponin response groups (Figure 3D). The observed reduction in pro-inflammatory cytokines (IL-1β, MCP-1) and fibrotic markers (α-SMA) in the saponin-treated group suggests attenuation of ROS-induced hepatic injury, consistent with the known antioxidant effects of red ginseng components.

### 3.5. RNA Sequencing and Gene Expression Analysis in High-Fat Diet-Induced Fatty Liver Mice

**Figure 4:**
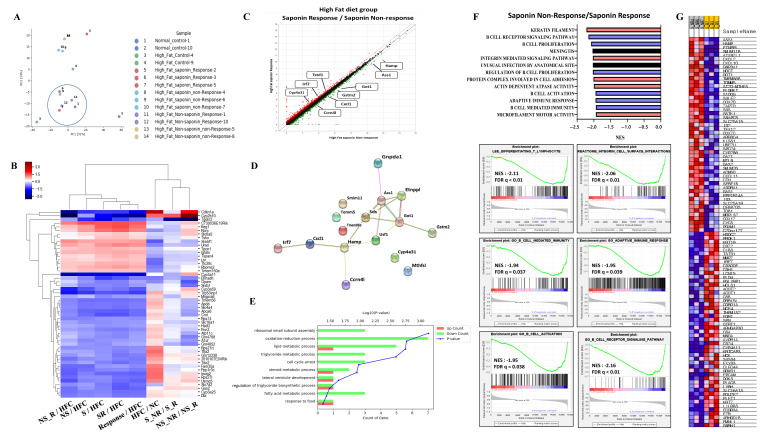
Transcriptomic analysis reveals molecular signatures differentiating saponin responders from non-responders in the High-Fat diet (HFD) group. (**A**) Principal component analysis (PCA) of RNA-seq data across normal control, HF control, saponin response, non-response, non-saponin response, and non-response groups. (**B**) Heatmap showing sample-to-sample correlations among the groups based on transcriptomic profiles. (**C**) Gene expression scatter plot comparing saponin response and saponin non-response groups; differentially expressed genes with fold change ≥ 1.5 and *p*-value ≤ 0.05 are highlighted. (**D**) STRING analysis of RNA-seq data comparing saponin response and saponin non-response mice, visualizing high-confidence protein–protein interactions (confidence score 0.7–1.0). (**E**) Gene ontology (GO) enrichment analysis comparing HFD and HFD + saponin groups, highlighting downregulation of pathways related to oxidation–reduction and lipid metabolism. (**F**) Gene set enrichment analysis (GSEA) identifying the top 20 enriched pathways in the saponin response group; a representative GSEA enrichment plot is shown below. (**G**) Heatmap showing key genes significantly enriched in the saponin response group compared to the non-response group, as derived from GSEA results.

Following the induction of fatty liver using a High-Fat diet, samples with an NAS greater than 3 at pre-biopsy were assessed. Cases with an NAS reduction of over 3 points at endpoint were classified as the response group, while those with an endpoint NAS above 6 were classified as the non-response group. RNA sequencing was conducted on 14 selected mice, including controls and both response and non-response groups for saponin and non-saponin treatments. PCA analysis revealed similarities between the normal control, High-Fat saponin response, and non-saponin response groups (Figure 4A). Heatmap analysis showed that gene expression changes in the High-Fat control were reversed in saponin and non-saponin treatments (Figure 4B). Differential expression analysis highlighted a reduction in the *Hamp1* gene in the saponin response group compared with that in the non-response group (*p* < 0.05, Figure 4C). STRING analysis of the saponin response group showed upregulation of *cyp4a31*, *Irf7*, and *Tstd1*, with several genes downregulated (Figure 4D). To further explore the biological processes affected by red ginseng saponin treatment, we performed GO enrichment analysis comparing HFD and HFD + saponin groups. Among the significantly enriched terms, “oxidation–reduction process,” “lipid metabolic process,” “triglyceride metabolic process,” and “fatty acid metabolic process” were notably downregulated in the saponin-treated group (Figure 4E). These findings suggest that red ginseng saponins ameliorate MASLD by suppressing oxidative and lipid metabolic stress, thereby contributing to improved hepatic homeostasis. GSEA indicated downregulation of B cell activity, T lymphocyte differentiation, and pathways related to adipogenesis and non-alcoholic fatty liver disease in the saponin response group compared to the non-response group (FDR < 0.25, Figure 4F). These findings suggest that saponins significantly influence immune modulation in responders, as illustrated by gene changes in the heatmap (Figure 4G). In particular, the downregulation of *HAMP1* and inflammatory signaling pathways in the saponin response group implies a reduction in oxidative stress, possibly mediated through both decreased iron availability and transcriptional regulation of antioxidant defenses.

### 3.6. Identification of Candidate Genes and Biomarkers from RNA Sequencing Data

A total of 16 genes were identified with a fold change greater than 1.5, normalized log2 values greater than 3, and a *p*-value less than 0.05. These genes include *Ass1*, *Ccrn4l*, *Cxcl1*, *Cyp4a31*, *Etnppl*, *Gnpda1*, *Got1*, *Gstm2*, *Hamp*, *Irf7*, *Mthfsl*, *Sds*, *Smim11*, *Tmem86b*, *Tomm5*, and *Tstd1*. To refine the selection of candidate genes, we applied more stringent criteria: fold changes greater than 2, normalized log2 values greater than 4, and a *p*-value less than 0.05. This analysis led to the identification of *Got1* and *HAMP1*. The *Got1* gene did not show a significant difference between the High-Fat control and the normal control. However, a significant reduction in *Got1* expression was observed in the High-Fat saponin response group compared to the High-Fat saponin non-response group. For the *HAMP1* gene, significant upregulation was noted in the High-Fat control compared with the normal control, and *HAMP1* expression was significantly reduced in the saponin response group compared to the saponin non-response group. Among the candidate genes with significant differential expression, liver-specific genes were selected as potential biomarkers. A scatter plot was generated using genes with a fold change of 1.5 or greater, a normalized log2 value of 3 or higher, and a *p*-value of 0.05 or less (Figure 4C). Based on the RNA sequencing data and GSEA analysis, *HAMP1* was selected as a potential biomarker for further investigation in subsequent experiments.

### 3.7. mRNA Expression and Histological Analysis of HAMP1 in Various Diet-Induced Liver Models

**Figure 5:**
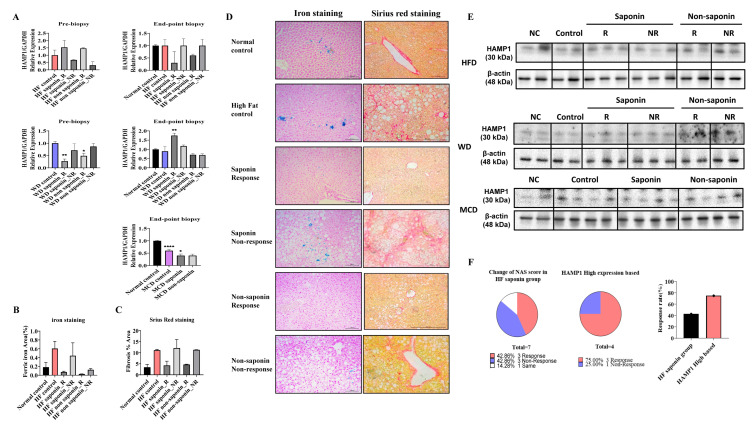
*HAMP1* expression correlates with iron overload and fibrosis severity in MASLD mouse models. (**A**) mRNA expression levels of *Hamp1* in liver tissues from pre-biopsy and endpoint samples in MASLD mouse models. (**B**) Quantitative graph representing the area of ferric iron accumulation based on liver iron staining. (**C**) Quantification of hepatic fibrosis using Sirius Red staining in the HFD model. (**D**) Representative liver sections from the HFD group stained for ferric iron (left) and Sirius Red (right) at the experimental endpoint. Scale bar = 100 µm. (**E**) HAMP1 protein expression assessed by Western blot analysis in MASLD mouse models. (**F**) Graph comparing NAS reduction rates before and after saponin administration in the HFD group, highlighting cases with concurrent Hamp1 upregulation and NAS improvement. Statistical significance is indicated by asterisks: * *p* < 0.05, ** *p* < 0.01, **** *p* < 0.0001.

In the pre-biopsy liver tissues of the HFD group, mRNA analysis revealed a significant increase in *HAMP1* expression, with a 1.538-fold rise in the High-Fat saponin response group compared to controls, while it decreased to 0.673-fold in the saponin non-response group. In the High-Fat non-saponin category, responders showed a 1.467-fold increase, but non-responders decreased to 0.314-fold. At the endpoint biopsy, *HAMP1* expression trended downward in both response groups. In the Western diet model, liver tissues were similarly analyzed. Pre-biopsy results showed a significant decrease in *HAMP1* expression in both saponin response (*p* = 0.0011) and non-saponin response groups (*p* = 0.0203). At the endpoint biopsy, *HAMP1* significantly increased in the saponin response group (*p* = 0.0021) compared with controls. The MCD diet model also showed a significant reduction in *HAMP1* expression in the MCD control (*p* < 0.0001) and a decrease in the MCD saponin group (*p* = 0.0479), suggesting different regulatory pathways for *HAMP1* based on MASLD induction (Figure 5A).

Representative liver sections from the HFD group stained for ferric iron (left) and Sirius Red (right) at the endpoint revealed increased ferric iron deposition in the High-Fat control group versus the normal control, while both the saponin and non-saponin response groups showed markedly reduced iron accumulation. Sirius Red staining also indicated reduced hepatic fibrosis in response groups, consistent with anti-fibrotic effects (Figure 5B–D). Western blot confirmed HAMP1 protein levels in MASLD mouse models (Figure 5E). In the High-Fat diet saponin group, the NAS reduction rate was 42.86% in responders and 75% in non-responders compared to the High-Fat control, indicating a 32.14% greater benefit from saponin response (Figure 5F). Moreover, reduced hepatic iron deposition observed in Prussian blue staining in conjunction with downregulated HAMP1 expression suggests attenuation of ROS generation via the Fenton reaction, providing further evidence of an antioxidant mechanism underlying the hepatoprotective effects of saponins.

### 3.8. Saponin-Associated Changes in Lipid Accumulation, ROS, and Fibrosis in Mouse and Human Liver Models

To explore the molecular effects of red ginseng saponins, we assessed inflammation-, oxidative stress-, and fibrosis-related markers in both HFD-fed mouse livers and human liver organoids exposed to lipotoxic stimulation.

**Figure 6:**
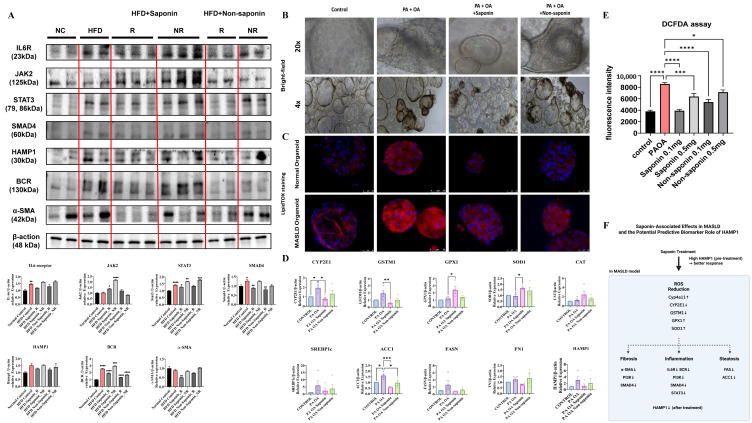
Saponin treatment alleviates lipotoxic injury and oxidative stress in patient-derived liver organoids. (**A**) Western blot analysis of inflammation and fibrosis-related proteins in liver tissues from HFD-fed mice treated with saponin or non-saponin fractions. (**B**) Bright-field images of patient-derived liver organoids treated with PA (200 μM) and OA (400 μM), along with either saponin or non-saponin fractions (100 μg/mL), captured at 4× and 20× magnification; scale bar: 100 μm. (**C**) LipidTOX staining reveals lipid accumulation in patient-derived liver organoids; scale bar: 50 µm. (**D**) mRNA expression of oxidative stress and metabolic genes (*CYP2E1*, *GSTM1*, *GPX1*, *SOD1*, *CAT*, *SREBP1c*, *ACC1*, *FASN*, *FN1*, *HAMP1*) in organoids under the same treatment conditions. Data are presented as mean ± SEM; each dot represents an individual organoid line. (**E**) DCFDA assay showing intracellular ROS levels in organoids. (**F**) Schematic model illustrating the key molecular pathways modulated by saponin treatment in MASLD. Solid arrows represent gene expression changes induced by saponin treatment, while dashed arrows indicate hypothesized connections to downstream phenotypes such as fibrosis, inflammation, and steatosis. Statistical significance is indicated by asterisks: * *p* < 0.05, ** *p* < 0.01, *** *p* < 0.001, **** *p* < 0.0001.

In the HFD-fed mouse model, Western blot analysis revealed that saponin treatment was associated with attenuation of key inflammatory and fibrotic signaling pathways in responder animals. While some inflammatory markers, such as IL6R and HAMP1, showed only modest changes, reductions in STAT3, SMAD4, BCR, and α-SMA levels were more prominent, suggesting a coordinated downregulation of pro-inflammatory and fibrotic responses (Figure 6A). These molecular patterns in saponin responders support the potential engagement of anti-inflammatory and anti-fibrotic mechanisms in vivo, although variability across individual markers highlights the need for further mechanistic validation. In liver organoids from normal and MASLD patients treated with saponin and non-saponin compounds alongside palmitic acid (PA) and oleic acid (OA), bright-field imaging showed darkened organoids in the PA + OA group, which improved significantly with saponin (Figure 6B). LipidTox staining revealed increased lipid levels in the PA + OA group compared with controls for both organoid types. After saponin treatment, lipid staining decreased, with a less pronounced effect in the non-saponin group (Figure 6C). To determine whether oxidative stress pathways were similarly affected in human models, we performed qRT-PCR on organoids treated with PA + OA and either saponin or non-saponin fractions. Saponin treatment significantly downregulated expression of key oxidative stress markers, including *CYP2E1*, *GSTM1*, and *GPX1*, while also upregulating antioxidant genes such as *SOD1*. Additionally, lipogenesis-related genes such as *ACC1* and *FASN* were suppressed, while *FN1* and *HAMP1* showed a decreasing trend in the responder group, suggesting concordance with murine data. These trends were generally consistent across four independent organoid lines, with individual sample values represented as dots (Figure 6D; Table 3). Intracellular ROS levels were assessed using a DCFDA assay in two independent organoid lines. PA + OA stimulation elevated ROS levels, which were subsequently reduced by both saponin and non-saponin fractions. Among them, saponin at 0.1 mg/mL most effectively restored ROS levels to near baseline (Figure 6E). These results suggest that saponins may help alleviate lipotoxic stress in liver organoids, at least in part, by modulating oxidative stress. Notably, the superior ROS-lowering effect of saponins over the non-saponin fraction highlights their stronger antioxidant capacity, reinforcing their therapeutic potential in redox-driven liver injury. A graphical summary of these mechanistic associations is presented in Figure 6F. These findings highlight the need for standardized *HAMP1* criteria, although further validation is needed due to limited sample size. These observations imply that red ginseng saponins may mitigate lipotoxic injury in liver organoids by reducing intracellular lipid accumulation and suppressing lipogenesis, likely through attenuation of oxidative stress induced by saturated fatty acids.

## 4. Discussion

In this study, we pursued three main objectives. First, we aimed to identify which components of red ginseng are effective in treating MASLD. Second, given the complex pathophysiology of MASLD, we sought to determine which specific MASLD subtypes exhibit therapeutic responses to red ginseng. Lastly, we aimed to identify biomarkers that could predict or monitor the maximum therapeutic efficacy.

To achieve these goals, we divided red ginseng into saponin and non-saponin fractions and administered them through MASLD-inducing diets. The MASLD models were induced using three different diets: MCD, HFD, and WD. Our results showed that saponins demonstrated significant hepatoprotective effects in both the WD and HFD groups. Previous studies have reported that saponins in red ginseng exert protective effects in MASLD by modulating lipid metabolism and reducing oxidative stress [5,22]. Several natural compounds such as curcumin, resveratrol, and berberine have been investigated for their antioxidant and anti-steatotic effects in MASLD models [23,24]. Compared with these, ginsenoside-based saponins may act via multiple pathways, including iron metabolism and immune signaling, though comparative studies are limited. Based on these findings, we hypothesized that saponins would be more effective in MASLD subtypes associated with obesity compared with lean MASLD types, such as the MCD group. Consistently, no therapeutic effect was observed in the MCD model, highlighting the importance of the metabolic context in evaluating treatment efficacy. However, considering the limitations of mouse models in replicating human metabolic responses, our primary focus remained on observing phenotypic changes in liver pathology. In parallel, we examined whether the Hamp1 gene, identified in mice, could serve as a biomarker using patient-derived organoids. Given that only one drug has been approved by the FDA for MASLD treatment, the significant reduction in inflammation observed in the WD group following saponin administration suggests the potential utility of saponins as therapeutic agents. In the HFD model, a distinct separation between responders and non-responders was observed, with NAS scores of 1.66 in responders versus 7 in non-responders.

We subsequently performed bulk RNA sequencing on the HFD group and identified genes with differential expression between the responder and non-responder subgroups. Candidate genes were selected using thresholds of >2-fold change, normalized log_2_ expression > 4, and *p*-value < 0.05. Among these, Got1 and Hamp1 emerged as key targets. Got1 expression did not differ significantly between the normal and High-Fat controls (fold change = 1.034), but it was notably reduced in the HFD saponin response group (fold change = 0.496). Similarly, Hamp1 expression was increased in the High-Fat control group (fold change = 2.104) relative to the normal group but markedly decreased in the saponin responder group (fold change = 0.328) compared with non-responders. This reduction led us to propose Hamp1 as a potential biomarker of saponin responsiveness. Notably, Hamp1 mRNA levels were elevated in pre-biopsy liver tissues of saponin responders but decreased in endpoint biopsies, suggesting treatment-associated modulation.

Previous studies have shown that Hamp1 is upregulated during inflammation, leading to decreased serum iron levels [25,26,27]. Excessive suppression of hepcidin can result in hepatic iron overload, contributing to oxidative stress [28]. Hepcidin, primarily produced by hepatocytes, regulates plasma iron levels by limiting intestinal iron absorption. It is also implicated in the pathogenesis of alcoholic liver disease, viral hepatitis, and autoimmune liver diseases. Among the various regulators of hepatic iron accumulation, Hamp1 plays a central role. Beyond iron metabolism, Hamp1 is involved in insulin resistance, lipid homeostasis, and inflammation [29]. Notably, the expression patterns of *CYP2E1*, *SOD*, and *GPX1* observed in mouse liver were largely recapitulated in human liver organoids following saponin treatment. This concordance supports the translational relevance of saponin response mechanisms across species. However, some differences in magnitude or baseline expression were observed, which may reflect species-specific metabolic and inflammatory regulation. Our findings align with previous reports showing increased hepcidin expression in fatty liver models. Moreover, the therapeutic effect of saponins was more evident in animals with elevated baseline Hamp1 expression, supporting the hypothesis that saponins may be particularly effective in MASLD subtypes associated with pronounced hepatic inflammation or insulin resistance.

Interestingly, the lack of therapeutic response in the MCD model, a lean MASLD subtype, emphasizes the need for subtype-specific approaches in MASLD therapy. These differential responses suggest that saponin efficacy may depend on the underlying metabolic and inflammatory state of the liver.

Transcriptomic analysis further revealed significant enrichment of genes involved in oxidation–reduction processes in the livers of saponin-treated mice, supporting a redox-regulatory role of ginsenosides in vivo. These findings are consistent with previous reports showing that specific ginsenosides, such as Rb1, Rd, and Rg1, can attenuate ROS accumulation and enhance antioxidant defenses. This is achieved by upregulating endogenous antioxidant enzymes, including *SOD1*, catalase (*CAT*), and *GPx*, in both in vitro hepatocyte models and in vivo fatty liver or diabetic mouse models [30,31]. This alignment between transcriptomic signals and prior experimental evidence reinforces the hypothesis that the hepatoprotective effects of ginsenosides are, at least in part, mediated by their capacity to mitigate oxidative stress.

Although we confirmed the differential expression of key oxidative stress-related and metabolic genes (*Cyp2e1*, *Gstm1*, *Gpx1*, *Sod1*, *Cat*, *Srebp1c*, *Fasn*, and *Fn1*) in liver organoids following saponin treatment at the transcript level, protein-level validation was not performed in this study. Furthermore, direct functional studies such as gene knockdown or overexpression of *Cyp2e1* or *Gstm1* were not conducted, which limits the mechanistic interpretation. Future studies incorporating protein validation and genetic perturbation will help to elucidate the causal role of these targets in mediating the effects of saponins.

These findings imply that saponins may confer dual antioxidant effects via both iron metabolism regulation and direct modulation of ROS-scavenging systems [9,10]. In addition to their antioxidant effects, saponins may exert protective roles in MASLD by modulating nuclear receptor signaling and metabolic gene expression. GO enrichment and transcriptomic analysis of saponin-treated mouse livers revealed significant regulation of genes involved in lipid metabolism, fatty acid oxidation, and oxidation–reduction processes. Notably, *Cyp4a11*, a gene regulated by the *PPARα* pathway and involved in fatty acid ω-hydroxylation, was upregulated in saponin responders, suggesting potential engagement of nuclear receptor signaling. In contrast, *Hamp* and *Got1*, which are linked to iron metabolism and cellular redox balance, were downregulated, possibly reflecting a reduction in oxidative and inflammatory stress.

In addition to the modulation of oxidative stress and lipid metabolism, our results suggest that red ginseng saponins may partially regulate immune signaling pathways. In particular, components of the IL6–JAK–STAT3 axis, known to contribute to inflammation and fibrosis in MASLD, showed differential expression patterns. *IL6R* expression tended to decrease in saponin responders, although this change was not statistically significant. Interestingly, JAK2 expression was slightly elevated, whereas STAT3 was significantly downregulated at both transcript and protein levels. This suppression of STAT3 was accompanied by decreased expression of downstream genes such as *HAMP1*, B cell markers, and fibrotic mediators (e.g., *α-SMA*). These findings, together with GO enrichment results showing reduced immune activation pathways (e.g., B cell proliferation and cytokine signaling), support the idea that saponins exert anti-inflammatory effects through partial or context-dependent inhibition of the IL6–JAK–STAT3 axis. The modest upregulation of JAK2 despite downstream inhibition may reflect complex regulatory dynamics, such as feedback regulation or post-transcriptional control. Further studies will be needed to clarify the specific regulatory nodes targeted by saponins.

Overall, our data support a model in which red ginseng saponins alleviate MASLD by dampening IL6–JAK–STAT3-mediated inflammation, which in turn reduces fibrosis, lipogenesis, and oxidative stress. The cross-validation of *HAMP1* downregulation in both murine and organoid systems suggests its potential as a biomarker linking inflammation and redox homeostasis. These multi-level effects highlight the therapeutic potential of saponins, although further mechanistic studies are warranted.

From a biomarker perspective, *Hamp1* may be useful for patient stratification. Previous studies have demonstrated that serum hepcidin levels are elevated in MASLD patients compared to healthy controls. A strong correlation has also been observed between hepatic *HAMP1* mRNA expression and serum hepcidin levels [32]. Given the regulatory role of hepatic hepcidin and iron metabolism in lipid homeostasis and MASLD progression [33], measuring *HAMP1* expression in blood or liver tissue may provide predictive insights into treatment responsiveness. Unlike clinically advanced MASLD therapies targeting FXR (e.g., obeticholic acid) or GLP-1 signaling (e.g., semaglutide), red ginseng-derived saponins may offer broader metabolic effects through iron homeostasis and redox regulation. However, the absence of pharmacokinetic, dose–response, and long-term efficacy data currently limits their translational readiness.

## 5. Conclusions

In conclusion, our findings suggest that MASLD patients with elevated pre-treatment HAMP1 expression may represent a subgroup that benefits most from saponin-based therapies. The observed reduction in HAMP1 levels following treatment correlates with histological improvement in preclinical models, suggesting a potential mechanistic and biomarker link. These results may inform future development of biomarker-guided treatment strategies, though clinical validation is still required. Future studies should explore the signaling pathways underlying saponin-mediated regulation of HAMP1, including clinical validation in human cohorts and mechanistic studies using knockout models. Given the central role of oxidative stress in MASLD progression, our findings also highlight the potential of red ginseng saponins as redox-modulating agents. The dual action of saponins through suppression of iron-induced ROS generation and activation of endogenous antioxidant defenses supports their investigation in future antioxidant-based treatment approaches.

## Figures and Tables

**Table 1:** Sequences of qRT-PCR mouse primers.

Gene	Primer
*Gapdh*	Forward: 5′-GTT-GTC-TCC-TGC-GAC-TTC-A-3′ Reverse: 5′-GGT-GGT-CCA-GGG-TTT-CTT-A-3′
*Col1a1*	Forward: 5′-CCT-CAG-GGT-ATT-GCT-GGA-CAA-C-3′ Reverse: 5′-CAG-AAG-GAC-CTT-GTT-TGC-CAG-G-3′
*Timp1*	Forward: 5′-ATC-TCT-GGC-ATC-TGG-CAT-CC-3′ Reverse: 5′-TTG-CAG-AAG-GCT-GTC-TGT-GG-3′
*Fn1*	Forward: 5′-CCC-TAT-CTC-TGA-TAC-CGT-TGT-CC-3′ Reverse: 5′-TGC-CGC-AAC-TAC-TGT-GAT-TCG-G-3′
*Mmp9*	Forward: 5′-GCT-GAC-TAC-GAT-AAG-GAC-GGC-A-3′ Reverse: 5′-TAG-TGG-TGC-AGG-CAG-AGT-AGG-A-3′
*Il1β*	Forward: 5′-CTC-ACA-AGC-AGA-GCA-CAA-GC-3′ Reverse: 5′-TCC-AGC-CCA-TAC-TTT-AGG-AAG-A-3′
*Srebp1c*	Forward: 5′-GAA-ACA-CTC-AGC-AGC-CAC-CA-3′ Reverse: 5′-CAA-GCT-TTG-GAC-CTG-GGT-GT-3′
*Fas*	Forward: 5′-CCC-TTT-TTG-AGG-AGG-CCA-AT-3′ Reverse: 5′-GCT-TCA-CGA-CTC-CAT-CAC-GA-3′
*Scd-1*	Forward: 5′-AGA-AGG-GCG-GAA-AAC-TGG-AC-3′ Reverse: 5′-AGG-CCG-GGC-TTG-TAG-TAC-CT-3′
*Mcp-1*	Forward: 5′-CAT-CAC-GGA-CAG-AGG-TTC-TGA-G-3′ Reverse: 5′-TCC-TCT-GTT-GTG-TGG-ATT-CAC-TC-3′
*Nlrp3*	Forward: 5′-TCA-CAA-CTC-GCC-CAA-GGA-GGA-A-3′ Reverse: 5′-AAG-AGA-CCA-CGG-CAG-AAG-CTA-G-3′
*Hamp1*	Forward: 5′-CAG-CAC-CAC-CTA-TCT-CCA-TCA-AC-3′ Reverse: 5′-CAG-ATG-GGG-AAG-TTG-GTG-TCT-C-3′

**Table 2:** List of antibodies used in this study.

Target	Supplier	Cat.no
α-SMA	Abcam, Cambridge, UK,	ab124964
PI3K	Abcam, Cambridge, UK,	ab86714
AKT	Abcam, Cambridge, UK,	ab8805
FN1	Abcam, Cambridge, UK,	ab2413
IL-1β	Abcam, Cambridge, UK,	ab254360
SREBP1	Abcam, Cambridge, UK,	ab3259
FAS	Abcam, Cambridge, UK,	ab82419
HAMP1	Abcam, Cambridge, UK,	ab190775
IL6R	Abcam, Cambridge, UK,	ab271042
JAK2	Cell signaling, Danvers, MA, USA	3230s
STAT3	Cell signaling, Danvers, MA, USA	9139s
SMAD4	Abcam, Cambridge, UK,	ab40759
BCR	Abcam, Cambridge, UK,	ab222406
β-actin	GENETEX, Irvine, CA, USA	GTX109639

**Table 3:** Sequences of qRT-PCR human primers.

Gene	Primer
*β-ACTIN*	Forward: 5′-AGG-AAG-GAA-GGC-TGG-AAG-AG-3′ Reverse: 5′-AGA-GCT-ACG-AGC-TGC-CTG-AC-3′
*HAMP1*	Forward: 5′-CTG-ACC-AGT-GGC-TCT-GTT-TTC-C-3′ Reverse: 5′-AAG-TGG-GTG-TCT-CGC-CTC-CTT-C-3′
*ACC1*	Forward: 5′-TTC-ACT-CCA-CCT-TGT-CAG-CGG-A-3′ Reverse: 5′-GTC-AGA-GAA-GCA-GCC-CAT-CAC-T-3′
*CYP2E1*	Forward: 5′-GAA-AAC-GAG-TGT-GTG-CTG-GA-3′ Reverse: 5′-CGG-GGA-ATG-ACA-CAG-AGT-TT-3′
*GSTM1*	Forward: 5′-TGA-TGT-CCT-TGA-CCT-CCA-CCG-T-3′ Reverse: 5′-GCT-GGA-CTT-CAT-GTA-GGC-AGA-G-3′
*GPX1*	Forward: 5′-GTG-CTC-GGC-TTC-CCG-TGC-AAC-3′ Reverse: 5′-CTC-GAA-GAG-CAT-GAA-GTT-GGG-C-3′
*SOD1*	Forward: 5′-CTC-ACT-CTC-AGG-AGA-CCA-TTG-C-3′ Reverse: 5′-CCA-CAA-GCC-AAA-CGA-CTT-CCA-G-3′
*CAT*	Forward: 5′-GTG-CGG-AGA-TTC-AAC-ACT-GCC-A-3′ Reverse: 5′-CGG-CAA-TGT-TCT-CAC-ACA-GAC-G-3′
*SREBP1*	Forward: 5′-TTC-GCT-TTC-TGC-AAC-ACA-GC-3′ Reverse: 5′-AAG-GAG-ACG-AGC-ACC-AAC-AG-3′
*FASN*	Forward: 5′-ATA-AGC-CCT-GTC-CTC-CAG-GT-3′ Reverse: 5′-TGG-AAG-AAA-AAT-GGG-CTT-TG-3′
*FN1*	Forward: 5′-CGG-TGG-CTG-TCA-GTC-AAA-G-3′ Reverse: 5′-AAA-CCT-CGG-CTT-CCT-CCA-TAA-3′

## Data Availability

The data presented in this study are available from the corresponding author upon reasonable request.

## Supplementary Materials

The following supporting information can be downloaded at: https://www.mdpi.com/article/10.3390/antiox14080943/s1, Figure S1: qRT-PCR and Western blot analysis of fibrotic, inflammatory, and lipogenic markers in liver tissues from MCD and Western diet mouse models.

## Abbreviations

ALT	Alanine aminotransferase
AST	Aspartate aminotransferase
MASLD	Metabolic dysfunction associated steatotic liver disease
HFD	High-fat diet
H&E	Hematoxylin & Eosin
GTT	Glucose tolerance test
GPX	Glutathione peroxidase
MC	Methylcellulose
MCD	Methionine and choline deficiency
NAFLD	Non alcoholic fatty liver disease
NAS	NAFLD activity scores
NASH	Non-alcoholic steatohepatitis
PBS	Phosphate-buffered saline
SR	Sirius red
SOD	Superoxide dismutase
TC	Total cholesterol
TG	Triglyceride
ROS	Reactive Oxygen Species
WD	Western diet
