# Supplementary material for: Biomarker-Driven Optimization of Saponin Therapy in MASLD: From Mouse Models to Human Liver Organoids
Source: Antioxidants (Basel). 2025 Jul 31;14(8):943. doi: 10.3390/antiox14080943 (PMC12383080; doi:10.3390/antiox14080943)
Supplement: Supplementary file 1 [file antioxidants-14-00943-s001.zip › antioxidants-3745084-supplementary.pdf]

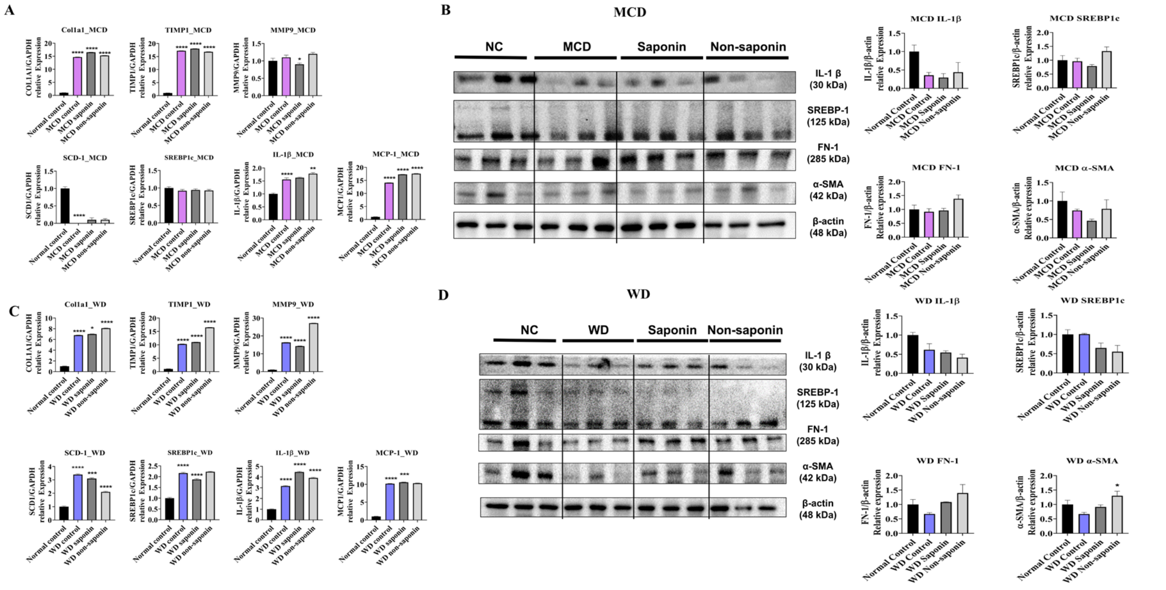

Figure S1: (A–D) qRT-PCR and Western blot analysis of fibrotic, inflammatory, and lipogenic markers in liver tissues from MCD and Western diet mouse models. Statistical significance is indicated by asterisks: * $p < 0.05$, ** $p < 0.01$, *** $p < 0.001$, **** $p < 0.0001$.
